# F140. HOMICIDES OF PHYSICIANS AND MENTAL HEALTH WORKERS

**DOI:** 10.1093/schbul/sby017.671

**Published:** 2018-04-01

**Authors:** Michael Knable

**Affiliations:** Sylvan C. Herman Foundation

## Abstract

**Background:**

Violence towards mental health care workers, and towards physicians in general, is a common occupational hazard. The goal of this work was to determine to what extent violence escalates to actual homicides, both for mental health workers and for physicians in general. Characteristics of the victims, the perpetrators and of the methods of homicide were examined in order to formulate recommendations for violence assessment and safety measures in healthcare settings.

**Methods:**

A systematic search for accounts describing homicides of mental health workers between 1981 and 2014, and for physicians between 1981 and 2017 was conducted. Cases of homicides committed by patients, family members of patients, and co-workers of the victims were included. Cases of homicide that occurred in correctional setting, or in agencies not focused on health care (such as child protective services) were excluded. News outlet accounts, internet sources, and the medical literature was searched for details of these cases. Data that were extracted included demographic details on victims and perpetrators, scene and method of homicide, presence of psychiatric diagnoses and prior treatment, and disposition of the perpetrators.

**Results:**

Results obtained for mental health workers has been published previously1. Thirty-three homicides of mental health workers were found and examined. Young women caseworkers who were unaccompanied during visits to residential treatment facilities were the most common victims. Men with a diagnosis of schizophrenia were the most common perpetrators. The most likely method of homicide was gunshot. Perpetrators often had a prior history of violence, criminal charges, involuntary hospitalization and nonadherence to medications. Thirty cases of homicides of physicians were found and examined. Psychiatry was the single most likely specialty of the victims (37%). Most homicides occurred in physician offices (33%). The most common psychiatric diagnosis of the perpetrators was schizophrenia (17%), but many other diagnoses were identified, and 33% of perpetrators could not be assigned a diagnosis. The most common method of homicide was again by gunshot.

**Discussion:**

Homicides of mental health workers, and of physicians generally, are rare events that emerge from a background of common aggression and violence in healthcare settings. Many of these homicides may have been preventable. Strategies to identify violence risk and to train acute care staff in possible prevention measures, as well as some policy and training measures will be discussed.

